# Correction: Li et al. Neutrophil Spatiotemporal Regulatory Networks: Dual Roles in Tumor Growth Regulation and Metastasis. *Biomedicines* 2025, *13*, 1473

**DOI:** 10.3390/biomedicines13123021

**Published:** 2025-12-10

**Authors:** Pengcheng Li, Feimu Fan, Bixiang Zhang, Chaoyi Yuan, Huifang Liang

**Affiliations:** 1 Hepatic Surgery Centre, Tongji Hospital, Tongji Medical College, Huazhong University of Science and Technology, Wuhan 430030, China; pcli9609@163.com (P.L.); feimu_fan@foxmail.com (F.F.); bixiangzhang@hust.edu.cn (B.Z.); 2 Hubei Key Laboratory of Hepato-Pancreato-Biliary Diseases, Wuhan 430030, China; 3 Key Laboratory of Organ Transplantation, Ministry of Education, NHC Key Laboratory of Organ Transplantation, Key Laboratory of Organ Transplantation, Chinese Academy of Medical Sciences, Wuhan 430030, China

**Text Correction**

The retracted reference 161 was deleted from the original article [1] along with the related content in the main text.

The following paragraph in Section 4.2.2, Paragraph 1 was deleted from the original article [1]:

“The nicotine-exposure further corroborates that early neutrophil infiltration into the lungs elevates the incidence of melanoma lung metastasis [161]”.

**References**

The retracted reference 161 was deleted from the original article [1]. With this correction, the order of some references has been adjusted accordingly. 

The authors state that the scientific conclusions are unaffected. This correction was approved by the Academic Editor. The original publication has also been updated.
